# The Effectiveness of Dipstick for the Detection of Urinary Tract Infection

**DOI:** 10.1155/2019/8642628

**Published:** 2019-10-23

**Authors:** Isaac Dadzie, Elvis Quansah, Mavis Puopelle Dakorah, Victoria Abiade, Ebenezer Takyi-Amuah, Richmond Adusei

**Affiliations:** ^1^Department of Medical Laboratory Science, University of Cape Coast, Cape Coast, Ghana; ^2^Department of Microbiology and Immunology, University of Cape Coast, Cape Coast, Ghana; ^3^Department of Biomedical Science, University of Cape Coast, Cape Coast, Ghana; ^4^Cape Coast Teaching Hospital, Cape Coast, Ghana

## Abstract

**Background:**

The balance between the choices of UTI diagnostic tools in most primary care settings has been settled for by the more rapid, less labour-intensive dipstick. This study aimed to evaluate the effectiveness of dipstick for diagnosing UTI.

**Method:**

A total of 429 urine samples were collected from patients suspected of UTI; cultured on cysteine-lactose-electrolyte-deficient (CLED) agar, blood agar, and MacConkey agar; and incubated at 37°C overnight. Urine cultures with bacteria count ≥10^5^ cfu/ml were classified as “positive” for UTI. A dipstick was used to screen for the production of nitrite (NIT) and leucocyte esterase (LE), following the manufacturer's instructions. Biochemical reactions of nitrite and leucocyte esterase > “trace” were classified as “positive.” A quantitative urine culture was used as the gold standard.

**Results:**

The highest sensitivity value and negative predictive value were recorded for the combined “NIT+ or LE+” dipstick results. The highest specificity value, positive predictive value, positive likelihood ratio, and negative likelihood ratio were recorded for “nitrite-positive and leucocyte esterase-positive” results. Combined “nitrite-positive or leucocyte-positive” result was relatively the best indicator for accurate dipstick diagnosis, with AUC = 0.7242. Cohen's kappa values between dipstick diagnosis and quantitative culture were <0.6.

**Conclusion:**

Combined performance of nitrite and leucocyte esterase results appeared better than the solo performance of nitrite and leucocyte esterase. However, little confidence should be placed on dipstick diagnosis; hence, request for quantity culture should be encouraged in the primary healthcare settings.

## 1. Introduction

Urinary tract infections (UTIs) constitute one of the most common infections often investigated in many clinical settings [1]. Bacteria, particularly *Enterobacteriaceae*, represent the leading cause of UTI [2]. UTI is associated with high morbidity and mortality, especially among high-risk subpopulation including pregnant women, children, and immunocompromised patients [2, 3]. Bacteria infect and colonise broad-spectrum areas of the urinary tract, including ureter, urethra, and the bladder. UTI clinically manifest as dysuria, urinary incontinence, and haematuria which could lead to kidney failure and hypertension [4].

Proper diagnosis of urinary tract infection is important to ensure prompt and accurate treatment of patients. The laboratory diagnosis of UTI hinges on a set of diagnostic assays including urine dipstick, biochemical test, microscopy, Gram staining, and quantitative urine culture. None of these diagnostic methods in itself is deemed sufficient for a sole diagnosis of UTI as their limitations are widely acknowledged [5]. To reduce the degree of errors of diagnosis, when any of the foregoing diagnostic methods are singly used, combining two or more of these methods appears to be the ideal diagnostic practice [5]. By doing so, it is imperative to include culture as a component of the selected set as it represents the gold standard for laboratory diagnosing of UTI. However, quantitative urine culture is laborious and requires a longer period to complete. Consequently, in most primary care settings, the use of a single (most often dipstick) or rarely two of these protocols without quantitative urine culture is often relied upon for clinical laboratory diagnosis of UTI [6, 7]. Urine culture is most often requested only when a patient is having a recurrent infection or when symptoms are quite severe.

Pyuria and bacteriuria are the key indicators of UTI. Nitrite and leucocyte esterase markers on the dipstick are used for the detection of pyuria and bacteriuria, respectively. Nitrite testing relies on the ability to convert nitrate to nitrite. Nitrite production is believed to be associated with the members of *Enterobacteriaceae* whereas other bacteria isolates such as *Staphylococcus saprophyticus*, *Pseudomonas* spp., and *Enterococcus* cannot produce nitrite from nitrate [8]. Another setback to the use of nitrite testing is the fact that it requires more than 4 hours for bacteria to complete the biochemical conversion of nitrate to nitrite and as such urine samples collected not more than 4 hours after patients have urinated are likely to yield unreliable results [5]. Leucocyte esterase relies on the ability of leucocytes to produce esterolytic protein that hydrolysis esters. Leucocyte esterase testing could produce false-positive results for patients having acute leukemia or patients on antibiotic treatment regimen [9]. Despite the known limitations, dipstick remains the most commonly used diagnostic test for diagnosing UTI in many primary healthcare settings. This study aimed to investigate whether dipstick testing could be solely relied upon for diagnosis of UTI using quantitative culture as the gold standard.

## 2. Materials and Methods

### 2.1. Sample Collection

The study collected midstream urine samples from patients suspected of UTI, who were directed by the physician to the laboratory for investigation at Cape Coast Teaching Hospital (CCTH), a tertiary healthcare facility in Ghana, from May to June 2019. CCTH is a major referral healthcare center that serves the populace of Cape Coast and its environs within the central region of Ghana. There was no gender or age restrictions on the participants included in the study. Clinical signs and symptoms were not taken into account. However, the study excluded patients who had used antibiotics in the prior week and patients who had used phenazopyridine in the prior 2 days. To avoid/reduce contamination, all the patients were first informed to wash their hands. They were also taught how to collect clean-catch midstream urine. Besides, all the female patients were informed to wash their genitals with a swab soaked in normal saline. Clean-catch midstream urine was collected in a sterile, wide-mouthed plastic capped bottle.

### 2.2. Dipstick Test

The dipstick test for the presence of nitrite and leucocyte esterase was conducted using Combur10-Test M-strip following the manufacturer's instructions (Roche, Canada). With reference to the manufacturer's guide for interpretation, dipstick testing that produced nitrite or leucocyte esterase result greater than trace was taken as positive.

### 2.3. Quantity Urine Culture Assay

Well-mixed urine samples were cultured on a plate containing approximately 25 ml of CLED agar (Oxoid, England), blood agar (Oxoid, England), and MacConkey agar (Oxoid, England) within 2 hours after collection using a 0.002-ml sterile loop. The plates were then incubated overnight at 37°C under aerobic conditions. Given the significant risks associated with the use of strict cut-offs, the standard agar-based clinical culture value of 10^5^ colony-forming unit (CFU)/mL was used to represent an arbitrary cut-off [10–12]. Thus, upon inspection, bacteria growth ≥10^5^ cfu/ml was taken a “positive” for UTI infection, whereas bacteria growth <10^5^ cfu/ml and mixed bacteria growth were taken as a contaminant. *Escherichia coli* ATCC 25299 was used as a quality control strain. Isolates were identified using Gram staining, morphological characteristics, and standard biochemical methods [13].

### 2.4. Statistical Analysis

Data were first recorded using Excel spread sheath and transferred onto Stata statistical software version 14.0 for analysis. The central tendency for age was presented as median (interquartile range). Diagnostic yield of nitrite or leucocyte esterase was calculated as the proportion of urine samples that were nitrite-positive or leucocyte esterase-positive, respectively. Two sample pretest was performed to find the difference in percentage yield by indexed test results (positive and negative) with 95% CI. Sensitivities, specificities, positive and negative predictive values, and positive and negative likelihood ratios were calculated for urine samples with 95% CI. Receiver operating characteristics curve (ROC) was used to generate area under curve (AUC) values to estimate the diagnostic accuracy of the indexed dipstick test [14]. All analysis was done at an alpha value of 0.05.

### 2.5. Ethical Consideration

The study was carried out with an approval clearance from Institutional Review Board Secretariat, protocol ID: UCC (UCCIRB/CHAS/2019/70). Additionally, an informed written consent was obtained from the study participants and confidentiality was kept.

## 3. Results

### 3.1. Characteristics of the Study Population

Of the 429 urine samples, 65 patients had urine culture <10^5^ cfu/ml, indicating positive urine culture. The remaining 364 showed either no significant growth (NSG) or no bacteria growth (NBG) with a urine culture. The median age of the patients was 39 years (interquartile range = 35). A proportion of 310 (72.3%) were females, whereas 119 (27.7%) were males.

### 3.2. Dipstick Diagnosis Results for Positive Urine Cultures

Nitrite-positive urine samples had substantially higher proportion of samples that were urine culture-positive 18/24 (75.0%, 0.532–0.902) compared with nitrite-negative urine samples 47/405 (11.6%, 0.087–0.151), yielding 62.7% (0.457–0.810, *P* < 0.001) more culture positives. Leucocyte esterase-positive urine samples 39/93 (41.9%, 0.317–0.526) also yielded 32.2% (0.215–0.428, *P* < 0.001) more culture-positive results compared with leucocyte esterase-negative urine samples 26/268 (9.7%, 0.064–0.139). *Escherichia coli* 41 (63.1%) was the most commonly isolated organism followed by *Citrobacter* spp. 17 (26.2%), *Enterobacter* spp. 4 (6.2%), *Serratia* spp. 2 (3.1%), and *Pseudomonas* spp. 1 (1.5%). Details of the results of positive UTI culture, nitrite, and leucocyte esterase results are provided in Table 1.

Using culture as the gold standard, the results for nitrite alone had a relatively low sensitivity of 27.7 (95% CI = 17.3–40.2). Positive predictive value (PPV) and negative predictive value (NPV) for nitrite alone were found to be 75.0 (95% CI = 53.3–90.2) and 88.4 (95% CI = 84.9–91.3), respectively. On the other hand, the lone performance of leucocyte esterase showed a sensitivity of 60.0 (47.1–72.0), specificity of 73.9 (95% CI = 69.1–78.3), and positive predictive value of 29.1 (95% CI = 21.6–37.7). The combination of nitrite-positive or leucocyte esterase-positive results yielded the highest sensitivity and NPV value of 72.3 (95% CI = 59.8–89.7) and 93.6 (95% CI = 90.1–96.2), respectively. On the other hand, the combination of nitrite-positive and leucocyte esterase-positive results yielded the lowest sensitivity 16.9 (95% CI 8.8–28.3) but the highest specificity value 99.7 (95% CI = 98.5–100) and PPV 91.7 (95% CI = 61.5–99.8). The results indicated a poor performance of dipstick in ruling out likely negative diagnostic culture, producing negative likelihood ratio (−LR) ranging from 0.38 (0.26–0.57) to 0.83 (0.75–0.93). Details on the performance of dipstick strip with urine culture as the gold standard are presented in Table 2.

**Figure 1:**
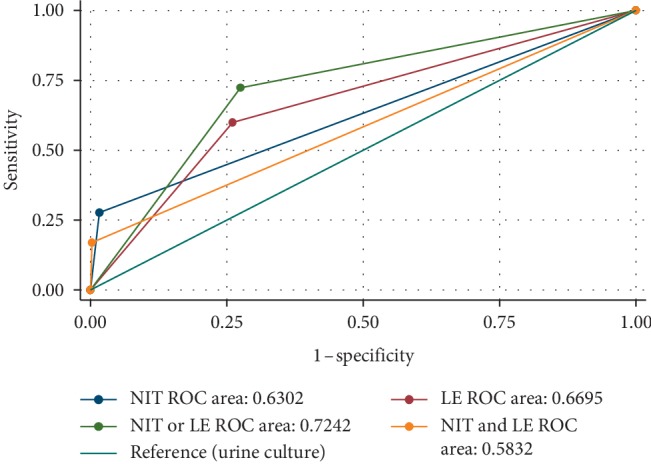
ROC curve for dipstick diagnosis with urine culture as a gold standard. NIT = nitrite, LE = leucocyte esterase.

ROC analysis showed that the combination of nitrite-positive or leucocyte esterase-positive was the best indicator of quantitative urine-positive or urine-negative culture, with a corresponding AUC value of 0.7242. This was followed by leucocyte esterase (AUC = 0.665), nitrite (AUC = 0.632), and nitrite-positive and leucocyte esterase-positive (AUC = 0.5832) in that order (Figure 1).

Of the 24 positive-nitrite results, 18 (4.2%) were true positive, whereas 6 (1.4%) were false positive compared with quantitative culture. Overall, nitrite results agreed with the quantitative culture results at 87.65% (0.222–0.481) with a kappa value of 0.351. Details on the agreement between nitrite and urine culture results are presented in Table 3.

Among 134 positive leucocyte esterase results, 39 (9.1%) were true positive, whereas 95 (22.1%) were false positive using quantitative urine culture as the gold standard. An agreement of 71.79% (0.142–0.330) and a kappa value of 0.233 were recorded between leucocyte esterase results and culture results (Table 4).

Comparing the results of “nitrite-positive or leucocyte esterase-positive” with quantitative urine culture, a proportion of 47 (10.9%) and 100 (23.3%) were recorded as true positive and false positive, respectively. The combination of nitrite-positive or leucocyte esterase-positive results agreed with quantitative urine culture at 72.49% with a corresponding kappa value of 0.295 (0.206–0.385) (Table 5).

Notably, there was very low false-positive 1 (0.2%) but high false-negative results 54 (12.6%) for “nitrite-positive and leucocyte esterase-positive” results (Table 6) with quantitative urine culture. Also, weak concordance [0.250 (0.127–0.374)] was observed between “nitrite-positive and leucocyte esterase-positive” results and quantitative culture.

## 4. Discussion

The present study assessed the diagnostic performance of urine dipstick, showing its potentials and limitations using quantitative urine culture as a reference test. Findings from this study showed that nitrite-positive and leucocyte esterase-positive urine samples relatively produce higher yields of urine culture-positive results than nitrite-negative and leucocyte esterase-negative urine samples respectively. This means nitrite-positive and leucocyte esterase-positive urine samples are arbitrarily expected to yield higher positive urine culture results than nitrite-negative and leucocyte esterase-negative samples respectively. However, the question that remains is to what extent can results produced with dipstick be relied upon in the absence of midstream urine culture?

From Table 2, it could be deduced that dipstick is relatively effective at diagnosing patients as negative who are truly negative than diagnosing patients as positive who are truly positive. This is because the observed specificity values were relatively higher than the observed sensitivity values (Table 2). As established, the presence of *Enterobacteriaceae* in the urinary tract invariably converts nitrate into nitrite. However, despite the fact that almost all isolates 64/65 (98.4%) recovered in this study belong to the family *Enterobacteriaceae*, performance of nitrite alone showed relatively low sensitivity 27.7 (95% CI = 17.3–40.2), similar to previous studies [15, 16] but approximately equal to that reported by Marques et al. [17] (sensitivity = 28. 0%). Likewise, the study by Prah et al. [15] and Marques et al. [17] was conducted among a generalized cohort population irrespective of age, gender, and symptoms using the same quantitative urine culture cut-off (10^5^ cfu/mL) as adopted by this present study. The low sensitivity value for nitrite could be explained by the fact that not all the isolates efficiently converted nitrate to nitrite or the patients may have passed out urine earlier before the sample collection, resulting in low nitrites concentration below detectable levels (although, participants were primed not to urinate before sample collection). In marked contrast, higher sensitivities for nitrite alone, leucocyte esterase alone, and “nitrite-positive and leucocyte esterase-positive” than their corresponding specificities have been reported in a previous study [18]. Perhaps, the difference in target population, exclusion and inclusion criterion, and sample size could argue for the observed discrepancies between the findings from the present study and those of Sirasaporn [18].

Conjunctive performance of nitrite and leucocyte esterase appeared relatively more reliable than the separate results from nitrite and leucocyte esterase as per the results of the present study. Evidently, “nitrite-positive or leucocyte-positive” results appeared to be the best index for distinguishing between positive and negative results for quantitative urine culture, which is similar to an earlier report [19]. The combined “nitrite-positive or leucocyte-positive” result seemed the most effective for identifying UTI-positive patients who are truly positive (sensitivity = 72.3%, 95% CI = 67.6–77.1), whereas combined nitrite-positive and leucocyte esterase results appeared to be very good at identifying UTI-negative patients who are truly negative (specificity = 99.7%, 95% CI = 98.5–100). Nonetheless, combined “nitrite-positive and leucocyte esterase-positive” results produced the lowest sensitivity. As argued by other investigators, there could be UTI without pyuria and also not all UTI infections are associated with inflammation (hence, no pus production) [20], explaining the low sensitivity observed for “nitrite-positive and leucocyte esterase-positive” results.

The ability of dipstick to predict negative results may be vital to preventing the risk of unnecessary initiation of antibiotic treatment [21, 22]. This is very important considering the increasing reports of antibiotic resistance worldwide. On the whole, NPVs recorded for both solo and combined dipstick markers were relatively high (88.4%–93.6%), suggesting dipstick can be a good predictor of negative results. Nitrite alone and leucocyte esterase alone showed NPV of 88.4% (95% CI = 84.9–91.3) and 91.2% (95% CI = 87.4–94.2), respectively. This means that inappropriate initiation of antibiotic treatment could be prevented in 88.4% of cases when nitrite is negative, whereas inappropriate initiation of treatment can be prevented in 91.2% of cases when leucocyte esterase is negative. The highest NPV recorded for “nitrite-positive and leucocyte esterase-positive” (93.6%, 95% CI = 90.1–96.2) means inappropriate initiation of treatment could be prevented in 93.6% cases when both nitrite and leucocyte esterase are negative. This finding concurs with the recommendation of the National Institute of Health and Care Excellence (NICE) which states that antibiotic treatment should not be started if both nitrite and leucocyte esterase are negative [22].

Nitrite alone recorded a relatively higher +LR 16.8 (6.93–40.72) which suggests it may be useful in ruling in UTI. Conversely, it has relatively low −LR 0.74 (0.63–0.86) indicating that it may not be a good indicator for ruling out UTI. Leucocyte alone appeared to be poor at both ruling in and ruling out UTI [+LR 2.30 (1.77–2.99), −LR 0.54 (0.40–0.73)]. The combination of “nitrite-positive and leucocyte-positive” results produced the highest +LR [61.6 (8.1–69.04)] suggesting that it may be the most useful index for ruling in UTI infection. This finding accords with a recent systematic review study that targeted children under the age of five years [23]. Notably, it appeared that +LR generally produced higher values relative to −LR, but the 95% CI was wider than −LR. This may insinuate the uncertainty of dipstick in ruling in UTI, which may be a reflection of the limited culture positives recorded by this present study.

Further, the present study investigated the level of agreement between nitrite and leucocyte esterase with urine culture. The agreement levels of nitrite and leucocyte esterase reported by this study were higher than those reported in Thailand [6]. In the present study, the highest agreement with urine culture was recorded for nitrite alone 87.65% (kappa = 0.351), followed by “nitrite-positive or leucocyte esterase results” 72.49 (kappa = 0.290). The recorded kappa values for nitrite alone (approximately = 0.4) and “nitrite-positive and leucocyte esterase-positive” (approximately = 0.3) depict “weak agreement” and “minimal agreement” with quantitative urine culture, respectively [24]. The <0.6 Cohen's kappa value recorded for all parameters suggests inadequate agreement of dipstick results with quantitative urine culture results, and consequently, little confidence should be placed on the result generated by dipstick. However, the limitations of the gold standard culture could argue for the disagreement observed between the two diagnostic assays [20].

This present study may have some limitations. First, there is no clearly accepted bacteria load cut-off for UTI in literature as varying values are adopted by different laboratories and institutions ranging from 10^2^ cfu/mL to 10^6^ cfu/mL [10, 11, 25, 26]. This may be consequential, especially in a more generalized cohort population. Supposedly, different thresholds would produce different results. However, this present study adopted an arbitral cut-off value of “10^5^ cfu/mL” that has been generally accepted and used for over 60 years [10], as cut-off bacterial load 10^3^ cfu/mL-10^4^ cfu/mL remains controversial [27]. Second, the relatively smaller sample size of culture positives may potentially affect the observed results and such results should be interpreted with caution.

## 5. Conclusion

The results from this study suggest that combined results from nitrite and leucocyte esterase produce better diagnostic performances compared with solo nitrite and leucocyte esterase performances. Also, dipstick appeared as a good predictor of negative urine cultures. Nonetheless, relative to quantitative urine culture, results produced by dipstick should not be overly relied upon for diagnosis. Therefore, quantitative urine cultures should be encouraged especially in the primary healthcare settings.

## Figures and Tables

**Table 1:** Causative bacteria isolate with nitrite and leucocyte esterase of quantitative urine culture results.

Organism	Nitrite-negative *n* (%)	Nitrite-positive *n* (%)	Leucocyte esterase-negative *n*(%)	Leucocyte esterase-positive *n*(%)	Total *n*(%)
*Escherichia coli*	26 (55.3)	15 (83.3)	15 (57.7)	26 (66.7)	41 (63.1)
*Citrobacter* spp	14 (29.8)	3 (16.7)	10 (38.5)	7 (17.9)	17 (26.2)
*Enterobacter* spp	4 (8.5)	0 (0)	1 (3.8)	3 (7.7)	4 (6.2)
*Serratia* spp	2 (4.3)	0 (0)	0 (0)	2(5.1)	2 (3.1)
*Pseudomonas* spp	1 (2.1)	0 (0)	0 (0)	1 (2.6)	1 (1.5)
Total	47 (100)	18 (100)	26 (100)	39 (100)	65 (100)

*n* = total number of isolates.

**Table 2:** Diagnostic performance of nitrite and leucocyte results relative to quantitative urine culture.

Culture	Sensitivity (%) (95% CI)	Specificity (%) (95% CI)	PPV (%) (95% CI)	NPV (%) (95% CI)	+LR (95% CI)	−LR (95% CI)
NIT+	27.7 (17.3–40.2)	98.4 (96.4–99.4)	75.0 (53.3–90.2)	88.4 (84.9–91.3)	16.8 (6.93–40.72)	0.74 (0.63–0.86)
LE+	60.0 (47.1–72.0)	73.9 (69.1–78.3)	29.1 (21.6–37.7)	91.2 (87.4–94.2)	2.30 (1.77–2.99)	0.54 (0.40–0.73)
NIT+ or LE+	72.3 (59.8–89.7)	72.5 (67.6–77.1)	32.0 (24.5–40.2)	93.6 (90.1–96.2)	2.63 (2.10–3.30)	0.38 (0.26–0.57)
NIT+ and LE+	16.9 (8.8–28.3)	99.7 (98.5–100)	91.7 (61.5–99.8)	87.1 (83.4–90.1)	61.6 (8.1–69.04)	0.83 (0.75–0.93)

NIT = nitrite, LE = leucocyte esterase, PPV = positive predictive value, NPV = negative predictive value, +LR = positive likelihood ratio, −LR = negative likelihood ratio.

**Table 3:** Concordance between nitrite results and quantitative urine culture results.

NIT	Culture					
	Positive	Negative	Agreement	Kappa	95% CI	*P* value
Positive	18 (4.2%)	6 (1.4%)	87.65%	0.351	0.22–0.48	*P* < 0.001
Negative	47 (10.9%)	358 (83.4%)				

NIT = nitrite.

**Table 4:** Concordance between leucocyte esterase results and quantitative urine culture results.

LE	Culture					
	Positive	Negative	Agreement	Kappa	95% CI	*P* value
Positive	39 (9.1%)	95 (22.1%)	71.79	0.24	0.14–0.33	*P* < 0.001
Negative	26 (6.1%)	269 (62.7%)				

LE = leucocyte esterase.

**Table 5:** Concordance between “nitrite-positive or leucocyte esterase-positive” results and quantitative urine culture results.

NIT or LE	Culture					
	Positive	Negative	Agreement	Kappa	95% CI	*P* value
Positive	47 (10.9%)	100 (23.3%)	72.49	0.30	0.21–0.39	*P* < 0.001
Negative	18 (4.2%)	264 (61.5%)				

NIT = nitrite, LE = leucocyte esterase.

**Table 6:** Concordance between “nitrite-positive and leucocyte esterase-positive” results and quantitative urine culture results.

NIT and LE	Culture					
	Positive	Negative	Agreement	Kappa	95% CI	*P* value
Positive	11 (2.5%)	1 (0.2%)	87.18	0.250	0.127–0.374	*P* < 0.001
Negative	54 (12.6%)	363 (84.6%)				

NIT = nitrite, LE = leucocyte esterase.

## Data Availability

All data generated or analyzed during this study are included in this published article.
